# The Importance of Myeloperoxidase in Apocynin-Mediated NADPH Oxidase Inhibition

**DOI:** 10.5402/2012/260453

**Published:** 2012-04-22

**Authors:** Ana Carolina de Almeida, Maria Marluce dos Santos Vilela, Antonio Condino-Neto, Valdecir F. Ximenes

**Affiliations:** ^1^Departamento de Química, Faculdade de Ciências, Universidade Estadual Paulista UNESP, CEP 17033-360, Bauru, SP, Brazil; ^2^Centro de Investigação em Pediatria (CIPED), Faculdade de Ciências Médicas, Universidade Estadual de Campinas, CEP 13083-887, Campinas, SP, Brazil; ^3^Instituto de Ciências Biomédicas, Universidade de São Paulo, CEP 05508-000, São Paulo, SP, Brazil

## Abstract

Apocynin is widely used as an inhibitor of the NADPH oxidase. Since myeloperoxidase (MPO) has been considered as essential for the mechanism of action of apocynin, here we used cells with different levels of MPO and compared their sensitivity to apocynin. HL-60 cells were differentiated with DMSO or IFN**γ**/TNF**α** and compared with peripheral mononuclear (PBMC) and polymorphonuclear cells (PMN). The relative MPO activity was PBMC = HL60 DMSO < HL60 IFN**γ** < PMN. Apocynin inhibited the intracellular reactive oxygen species production by PMN (80%) and IFN**γ**/TNF**α**-differentiated HL-60 cells (45%) but showed a minor effect in PBMC and DMSO differentiated HL-60 cells (20%). The addition of azide decreased the efficiency of apocynin in PMN and the addition of peroxidase increased the inhibition in PBMC. We also determined the gene expression of the components gp91phox, p47phox, p22phox and p67phox in the resting cells. Apocynin did not change gp91phox, p47phox or p22phox gene expression in nonstimulated PBMC, HL60 DMSO, HL60 IFN**γ**/TNF**α**, and PMN and has a subtle increase in p67phox in HL60 IFN**γ**/TNF**α**. The results from this work suggest that a rational search for better inhibitors of NADPH oxidase in leukocytes should include a correlation with their affinity as substrates for MPO.

## 1. Introduction

NADPH oxidases (NOXs) comprise a family of multicomponent enzymatic systems that catalyze the reduction of molecular oxygen to superoxide anion radical [1–3]. The best characterized and studied member of the NOX family is NOX2, which is expressed in phagocytic cells and has its primordial microbicidal function in the innate immune system as the primary source of reactive oxygen species (ROS). NOX2 is comprised by cytosolic factors p47phox, p67phox, and p40phox and the membrane-linked p22phox and gp91phox factors, which are the catalytic subunit. NOX2 also has a regulatory GTPase, Rac2 (in neutrophils), or Rac1 (in monocytes) [4, 5]. NOXs, including NOX2, are also expressed in endothelial cells and have direct influence in the oxidative imbalance, which is considered a pivotal event in the endothelial dysfunction and, consequently, in the initiation and/or progression of chronic inflammatory and vascular diseases [5]. Hence, the inhibition or modulation of NOXs activities may have direct benefit in the treatment of these pathologies [5].

Apocynin (4-hydroxy-3-methoxyacetophenone) is widely used as an inhibitor of NOXs and of the concomitant ROS production in experimental models involving phagocytic [6–8] and nonphagocytic cells [9–13]. The effects of apocynin include the reduction of p47phox expression and membrane translocation [14], inhibition of NF-*κ*B activation and expression [14], interference in arachidonic acid metabolism [15], inhibition of phagocytosis of opsonized microorganisms [16], inhibition of HOCl production by phagocytes [16], and reduction of cytokine release by monocytes and T cells [7, 15–17].

There is evidence of the role of the neutrophil peroxidase, myeloperoxidase (MPO) in the inhibitory mechanism of apocynin in phagocytic cells [9, 18]. However, the effect of apocynin in nonphagocytic cells, which do not contain MPO, is still controversial. Some authors suggest that in nonphagocytic cells apocynin does not inhibit NADPH oxidase system but acts as an antioxidant [12] or even as stimulator of ROS production [10, 11], and others suggest that apocynin and its dimer do inhibit NADPH oxidase system even in the absence of MPO [9, 13]. Here, we addressed these questions using cells with different content of MPO and comparing the inhibitory potency of apocynin. Additionally, we evaluated if the apocynin effect on NOX2 could be related to a regulation of gene expression for its components Gp91phox, p47phox, p22phox, and p67phox in nonstimulated cells.

## 2. Materials and Methods

### 2.1. Chemicals

Apocynin, phorbol 12-myristate 13-acetate (PMA), dihydrorhodamine 123, paraformaldehyde, zymosan, azide, 3,3′,5,5′-tetramethylbenzidine (TMB), lucigenin, dextran, horseradish peroxidase (HRP), and RPMI-1640 Medium were purchased from Sigma-Aldrich (St. Louis, MO, USA). Ficol-Paque was purchased from GE Healthcare. Red blood cell (RBC) lysis buffer was purchased from eBioscience. Hydrogen peroxide was prepared by diluting a 30% stock solution and calculating its concentration using its absorption at 240 nm (*ϵ*240 nm = 43.6 M^−1^ cm^−1^). Apocynin stock solution was prepared by dissolving the compound in dimethyl sulfoxide (DMSO). PMA was dissolved in DMSO at 10 *μ*g/mL. TMB solution was prepared dissolving 10 mM TMB and 100 *μ*M potassium iodide in 50% dimethylformamide and 50% acetic acid (800 mM) (v/v). Human serum-opsonized zymosan was prepared as described [19] in a final concentration of 10 mg/mL.

### 2.2. Culture and Differentiation of HL-60 Cells

HL-60 cells were obtained from ATCC and kept in culture in RPMI 1640 medium supplemented with 10% fetal bovine serum, 2.0 mM *L*-glutamine, 10 U/mL penicillin, and 100 *μ*g/mL streptomycin, in humidified atmosphere, with 5% CO_2_ at 37°C. HL-60 differentiation was induced by 1.3% DMSO or 200 U/mL IFN-*γ* and 1000 U/mL TNF-*α*. Cells were cultured during 5 days, and the medium was replaced every 2 or 3 days. Cell differentiation was accompanied by cytochemical test to monitor peroxidase-positive granules, and microscopic indicators of cell differentiation, like reduction of the nucleus:cytoplasm ratio and changes in the pattern of azurophilic granules. After differentiation, cells were incubated with 100 *μ*M or 1.0 mM apocynin during 15 minutes, 2 or 4 hours.

### 2.3. Isolation of Human Neutrophils and Peripheral Blood Mononuclear Cells

Polymorphonuclear (PMN) and peripheral blood mononuclear cells (PBMCs) were separated by Ficoll-Paque (1.119 and 1.077 g/mL, resp.) density gradient centrifugation from 20 mL of blood from healthy donors (*n* = 6 for each experiment) [20]. After isolation, the cells were resuspended in phosphate buffered saline (PBS) supplemented with 1.0 mM calcium chloride, 0.5 mM magnesium chloride, and 1.0 mg/mL glucose (supplemented PBS) or RPMI-1640 supplemented with 10% fetal bovine serum, 2.0 mM L-glutamine, 100 U/mL streptomycin, and 100 U/mL penicillin for longer culture periods. Flow cytometry assays were developed with total blood or total leukocytes in which erythrocytes were lysed with RBD lysis buffer according to the needs of the evaluation.

### 2.4. Determination of MPO Activity in Leukocytes and Differentiated HL-60 Cells

The determination of MPO activity in the different cell populations studied was based on TMB oxidation. 400 *μ*L of phosphate buffer 100 mM pH 5.4 and the cationic detergent cetrimide 0.3% were added to 1.0 × 10^6^ cells. The cells were lysed by sonication. Samples were centrifuged (10.000 rpm) and the pellet discarded. In a 1.0 mL cuvette, 150 *μ*L of the supernatant, 745 *μ*L of phosphate buffer, and 80 *μ*L of tetramethylbenzidine (TMB) in dimethylformamide (DMF) were homogenized, and 25 *μ*L of H_2_O_2_ 12 mM was added at the moment of the reading, what was performed at 655 nm and took place every 1 minute during 30 minutes [21].

### 2.5. Cytometry Assay (Intracellular ROS)

Dihydrorhodamine 123 (DHR) is largely used in the detection of intracellular oxidant species production by cell systems. DHR oxidation by ROS results in the formation of rhodamine, a highly fluorescent component. Total leukocytes were incubated with apocynin (1.0 mM) for 2 h and then stimulated with PMA (400 nM) for 10 min. After PMA stimulation, cells were incubated with DHR (10 mg/mL) for 5 min, washed once with PBS, and suspended in PBS/BSA/azide buffer. Fluorescence of gated PMN was detected at FL1, counting 30,000 events/gate, in an FACS Canto flow cytometer (BD, Franklin Lakes, NJ, USA). Data was analyzed using the Flowjo Flow Cytometry Analysis Software (Treestar Inc., Ashlan, OR, USA), and results were recorded as fluorescence intensity and percentage of positive cells in the sample.

### 2.6. Chemiluminescence Assay (Superoxide Anion Radical)

Lucigenin-dependent chemiluminescence is a sensitive, reproducible, and reliable technique for superoxide detection. In this reaction, superoxide reduces lucigenin to its cation radical, which reacts with a second superoxide anion to form the energy-rich dioxetane molecule emitting a photon [22]. In our assay, PMN cells and PBMC (1.0 × 10^6^ cells/mL) were preincubated with apocynin (100 *μ*M) for 15 min in supplemented PBS. Next, lucigenin (5.0 *μ*M) and opsonized zymosan (1.0 mg/mL) were added, and the light emission was measured for 30 min at 37°C (Centro Microplate Luminometer LB960, Berthold Technologies, TN, USA). The integrated light emission was used as an analytical parameter. The inhibitory potency was calculated by accounting for the light emission generated by the control, in which the cells were incubated in the absence of the tested compounds.

### 2.7. Gene Expression Assay

Gp91phox, p47phox, p22phox, and p67phox gene expression was performed by real-time PCR. This methodology is based on monitoring the fluorescence emitted during the polymerizing chain reaction (PCR) by the binding of a fluorescent dye (SYBR Green) at the newly synthesized strand. Briefly, RNA was extracted from treated cells using Trizol Kit (Invitrogen, Catalog no. 15596-026), and reverse transcription reaction was performed using the Superscript II RT Kit (GIBCO BRL, Gaithersburg, MD) and random hexamers. Primers specific for the target genes were designed using the Primer Express Software (Applied Biosystems, Foster City, CA, USA), and *β*-actin gene expression was used as standard control. Real-time PCR was performed in duplicate using the SYBR Green Master Mix (Applied Biosystems) in a 7500 Sequence Detector System (Applied Biosystems). In order to confirm PCR specificity and reproducibility, intra-assay precision was calculated according to the equation *E* = 10^(1−1/slope)^. 

### 2.8. Statistical Analysis

Results were presented as median with range. Comparisons among samples treated with apocynin and control samples (incubated with the vehicle) were made using the Mann-Whitney test for unpaired data. Results were considered significant with a *P* value <0.05 [23]. 

## 3. Results

### 3.1. MPO Activity versus Inhibition of ROS by Apocynin

**Figure 1:**
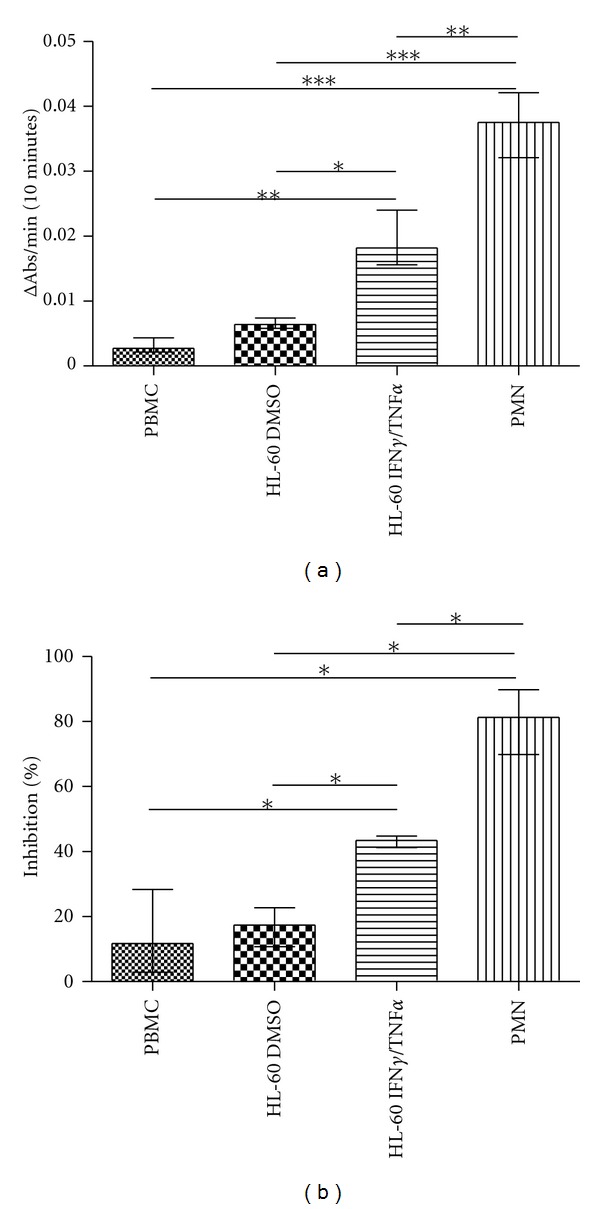
MPO activity (a) and the effect of apocynin (1.0 mM for 2 hours) on ROS production (b) by PBMC, DMSO-differentiated HL-60 cells, IFN-*γ*/TNF-*α*-differentiated HL-60 cells, and PMN. Percentage of inhibition was calculated in comparison to the cells incubated with the vehicle (DMSO). Data represents at least four separated experiments (*P < 0.05, **P > 0.01, ***P < 0.005, Mann-Whitney test).

HL-60 cells were differentiated with 1.3% DMSO or 100 U/mL IFN-*γ* and 1000 U/mL TNF-*α* during 5 days. This procedure resulted in two populations with different levels of MPO (Figure 1(a)). The MPO activity of differentiated HL-60 cells was also compared with leukocytes obtained from the blood of healthy donors. We found that PBMC and DMSO-differentiated HL-60 cells presented the same level of MPO activity. PMN cells showed MPO activity increased even when compared to IFN-*γ*/TNF-*α*-differentiated HL-60 cells. In resume, the following crescent order for MPO activity was observed: PBMC = HL60 DMSO < HL60 IFN*γ*/TNF*α* < PMN. In the sequence, the cells were activated with PMA, and the inhibitory potency of apocynin was measured. Apocynin strongly inhibited the intracellular ROS production by PMN cells (around 80%) and IFN-*γ*/TNF-*α*-differentiated HL-60 cells (around 45%) but showed a subtle effect in PBMC and DMSO-differentiated HL-60 cells (around 20%) (Figure 1(b)). MPO is relevant for apocynin mechanism, since apocynin inhibitory effect was increased in cells with increased MPO activity. 

### 3.2. Effect of HRP and Azide on NADPH Oxidase Inhibition by Apocynin

**Figure 2:**
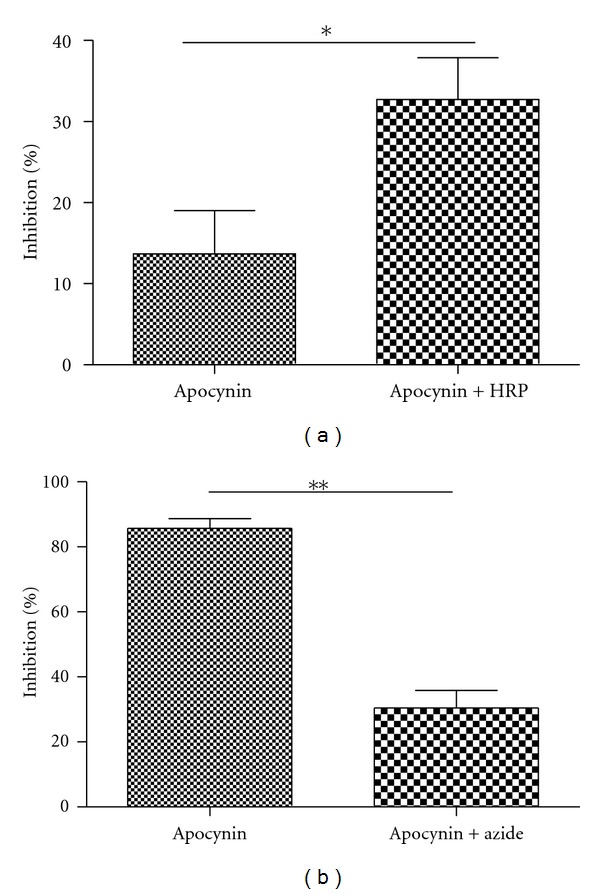
The effect of HRP and azide on apocynin inhibition of superoxide anion production by PBMCs and PMN. (a) PBMC were preincubated with 100 *μ*M of apocynin and 400 nm of HRP for 15 min. (b) PMN cells were preincubated with 100 *μ*M of apocynin and 5.0 mM of sodium azide for 15 min. Percentage of inhibition was calculated in comparison to the control group (absence of apocynin). Data represents at least four separated experiments (*P < 0.05, **P < 0.01, Mann-Whitney test).

In order to confirm the role of MPO on apocynin mechanism of action, we pharmacologically simulated an increase in peroxidase activity by adding HRP to the PBMC. The cells were incubated with apocynin (100 *μ*M) and HRP (400 nM) during 15 minutes and then stimulated with opsonized zymosan. Superoxide anion detection was performed by chemiluminescence during 30 minutes. HRP significantly increased the inhibitory effect of apocynin on superoxide anion release (Figure 2(a)). Additionally, we evaluated the apocynin effect on superoxide anion release in PMN incubated with the MPO inhibitor, azide. The cells were incubated with apocynin (100 *μ*M) and sodium azide (5.0 mM) during 15 minutes, and superoxide production was stimulated by opsonized zymosan (10 *μ*g/mL). Azide significantly reversed the inhibitory effect of apocynin on superoxide anion release (Figure 2(b)).

### 3.3. Effect of Apocynin on Components of NADPH Oxidase Gene Expression and the Role of MPO

**Figure 3:**
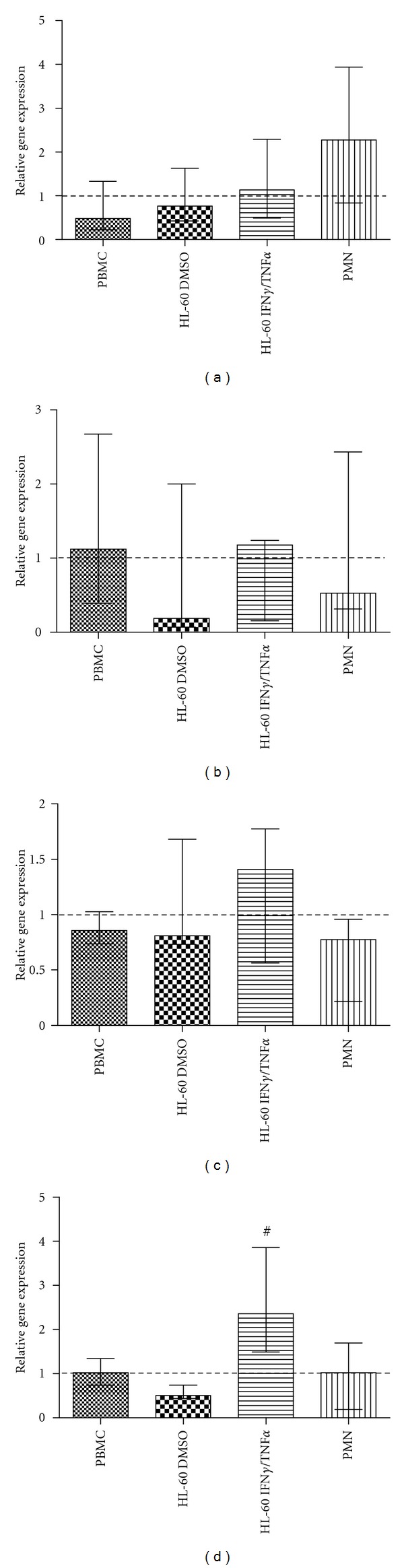
The effect of apocynin on the gene expression of gp91phox (a), p47phox (b), p22phox (c), and p67phox (d) by leukocytes. PBMC, PMN, and differentiated HL-60 cells were incubated with 1.0 mM of apocynin during 4 hours, RNA was extracted, and gene expression was accessed by real-time PCR. Relative gene expression was calculated considering one PBMC control sample as reference. Data represents at least four separated experiments (^#^ *P* < 0.05, Mann-Whitney test).

In the sequence, we evaluated if the apocynin effect on NADPH oxidase activity could be related to a regulation of NADPH oxidase gene expression. For this purpose, we determined gene expression of the components gp91phox, p47phox, p22phox, and p67phox of NADPH oxidase by resting cells incubated with apocynin. PBMC, PMN, and differentiated HL-60 cells were incubated with apocynin during 4 hours, and gp91phox, p47phox, p22phox, and p67phox were determined using real-time PCR. Apocynin did not change gp91phox, p47phox or p22phox gene expression in PBMC, HL60 DMSO, HL60 IFN-*γ*/TNF-*α*, and PMN cells (Figures 3(a), 3(b), and 3(c)), but increased p67phox gene expression in HL60 IFN-*γ*/TNF-*α* (Figure 3(d)).

## 4. Discussion

 The major oxidant system in leukocytes is constituted by NADPH oxidase and MPO, which are the key enzymes in a cascade of reaction leading to ROS as H_2_O_2_, hypochlorous acid (HOCl), hypobromous acid (HOBr), and hypothiocyanous acid [24–26]. In this concern, these enzymes are target in the development of new drugs for treatment of chronic inflammatory pathologies. Apocynin is one of these drugs for which many attentions have been given in the last few years. Curiously, the mechanism of action of apocynin involves both MPO and NADPH oxidase, since its oxidation catalyzed by MPO seems crucial for the inhibition of NADPH oxidase via formation of a dimeric oxidation product and/or the generation of a transient pro-oxidant apocynin radical. In the first case, there is evidence that the dimeric product is more potent than apocynin itself [9], or in other words, apocynin could be assigned as a prodrug. In the second proposal, the pro-oxidant apocynin radical could oxidize essential sulfhydryl residues in the components of NADPH oxidase, leading to its inactivation [27]. In a recent paper, the importance of the oxidation of apocynin was reinforced, since apocynin-derived oligophenols were still more potent than apocynin in endothelial cells where MPO is not expressed [28]. Despite the exact mechanism, the fact is that the oxidation products or yet the transient species generated during the oxidation apocynin inside the cells impede the migration of the component p47phox to the membrane avoiding the NADPH assembly [14]. In agreement with that, here we have confirmed the importance of MPO in the inhibition process of apocynin. However, more than just confirmed, we obtained a direct relationship between the cellular MPO level and the inhibition of ROS generation using differentiated promyelomonocytic HL-60 cells and peripheral blood leukocytes. This dependence of MPO is not an isolated case. Indeed, MPO-mediated oxidation is part of the mechanism of activation or metabolism/deactivation of several drugs. For instance, MPO mediated the oxidation of nimesulide, carbamazepine, and mitoxantrone leading to the formation of even more active products [29–31]. The oxidation of doxorubicine, daunorrubicine, and *β*2 adrenergic agonists by MPO inactivates the drugs, turning them nontoxic to MPO-rich cells [32].

 Besides the activation/inactivation of its cytosolic components, the enzymatic activity of the NADPH oxidase system can be also altered at transcriptional level by phytochemicals. For instance, berberine, an alkaloid traditionally used in Chinese Ayurvedic and North American medicine to the treatment of diarrhea [33], reduces gp91phox expression in LPS-stimulated macrophages, an effect that can be related to its anti-inflammatory and antiproliferative properties. Polyphenols, abundant in tea, cocoa, and red fruits, and known by its anti-inflammatory, antitrombotic, anti-ischemic, antioxidant, and vasorelaxing properties, reduce p22phox, gp91phox and p47phox, and NADPH oxidase activity in phagocytes [30]. Apocynin also have this property in stimulated cells. For instance, the reduction of gp91phox gene expression in LPS-stimulated PBMC [34]; decreased p47phox and gp91phox in rat kidney and aorta treated with aldosterone [7]; in PMA-differentiated THP-1 cells apocynin potentiates testosterone-induced inhibition of p47phox gene expression [35]. Here, we found that apocynin did not change gp91phox, p22phox, and p47phox in all cell populations studied but slightly increased p67phox gene expression in IFN-*γ*TNF-*α*-differentiated HL-60 cells. We also observed that, unlike the inhibition of NADPH oxidase activity, the apocynin effect on NADPH oxidase gene expression in resting cells is not related to the level of MPO activity. This difference amongst our results and the literature can be due to the activation state of the studied cells, since we evaluated apocynin effect in differentiated, but not stimulated cells.

In conclusion, our results shed some light on apocynin mechanism of action. We have demonstrated that MPO activity is a key factor for apocynin effect on NADPH oxidase activity, but this concept does not extend to the NADPH oxidase transcriptional regulation in nonstimulated cells. Considering the role of MPO in the inhibitory mechanism of apocynin and its increasing application as anti-inflammatory drug, the results from this work suggest that a rational search for better inhibitors of NADPH oxidase in leukocytes should include a correlation with its affinity as a substrate for MPO. This property could provide a higher selectivity as NADPH oxidase inhibitor.
